# Metabolically Doping of 3D Diatomaceous Biosilica with Titanium

**DOI:** 10.3390/ma15155210

**Published:** 2022-07-27

**Authors:** Weronika Brzozowska, Myroslav Sprynskyy, Izabela Wojtczak, Przemysław Dąbek, Michał J. Markuszewski, Andrzej Witkowski, Bogusław Buszewski

**Affiliations:** 1 Institute of Marine and Environmental Sciences, University of Szczecin, Mickiewicza 16, 70-383 Szczecin, Poland; weronika.brzozowska@phd.usz.edu.pl (W.B.); przemyslaw.dabek@usz.edu.pl (P.D.); andrzej.witkowski@usz.edu.pl (A.W.); 2 Institute of Marine and Environmental Sciences, Doctoral School, University of Szczecin, Mickiewicza 16, 70-383 Szczecin, Poland; 3 Department of Environmental Chemistry and Bioanalytics, Faculty of Chemistry, Nicolaus Copernicus University in Torun, 7 Gagarina Str., 87-100 Torun, Poland; izabelawojtczak1991@gmail.com (I.W.); bbusz@umk.pl (B.B.); 4 Department of Biopharmaceutics and Pharmacodynamics, Faculty of Pharmacy, Medical University of Gdansk, 80-210 Gdansk, Poland; michal.markuszewski@gumed.edu.pl; 5 Centre for Modern Interdisciplinary Technologies, Nicolaus Copernicus University, Wilenska 4, 87-100 Torun, Poland

**Keywords:** diatoms, diatomaceous biosilica, metabolic inserting, titanium doped biosilica

## Abstract

Diatoms represent, in terms of species number, one of the largest groups of microalgae that have the ability to synthesize phenomenal mineral composites characterized by complex hierarchical structures. Their shells, called frustules, create intricately ornamented structures, reminiscent of the most sophisticated, natural mosaics. Ordinated pore systems perforate siliceous walls of the frustules with diameters ranging from nano to micro-scale, forming openwork three-dimensional silica structures. The use of these features is one of the main challenges in developing new technological solutions. In this study we assess the ability of selected diatom species (*Pseudostaurosira trainorii*) for metabolic insertion of soluble titanium from the culture medium into the structure of amorphous silica cell walls by its cultivation in laboratory conditions. The study is aimed at obtaining new and strengthening the already existing optical properties of diatomaceous biosilica. The physicochemical properties of the obtained materials have been studied using a series of instrumental methods.

## 1. Introduction

The increased velocity with which various types of micro- and nanostructures began to appear in the process of creating all kinds of devices for advanced modern technologies is phenomenal [1]. Nevertheless, the main barrier limiting the velocity of prototype device development is the fabrication of materials with a three-dimensional structure, which is often necessary to achieve the required performance and functionality [2]. In the search for a solution to create functional, three-dimensional biocomposites with a defined hierarchical structure, many biologists, chemists and physicists have turned their attention to microorganisms [3,4,5]. At the micro- and nano-scale the sophistication and functionality of biological structures are significantly increased over equivalent human-made devices [6,7]. For this reason, the unique properties of diatoms led in 1988 to the formation of a new interdisciplinary research area called diatom nanotechnology [8]. Since then, the distinctive ability of these single-celled microalgae to form siliceous shells with precise, hierarchical 3D structures has attracted so much scientific interest that it has made these biological, openwork structures a possible building substrate for a new generation of micro- and nanodevices [3,9,10,11,12,13,14,15].

The size and morphology of diatoms are strictly species specific, and their shells, known as frustules, show a diversity of ornamentation. These “living opal” and “crystal palaces” [16,17], as these microalgae are referred to, due to their perfectly ordered, three-dimensional structure, unique optical properties, thermal and mechanical stability, and biocompatibility, can be a valuable resource for applications in modern technologies as a raw material for the production of optoelectronic devices, biosensors, gas sensors, biocatalysts, adsorbents, efficient filters, semiconductors, solar cells, or carrier materials for active pharmaceutical substances [4,18,19]. Diatomaceous biosilica is already receiving significant interest as a raw material for commercial applications. Recently, researchers have extensively investigated the potential of frustules as a substrate for the production of natural electrodes suitable for energy storage and production [20]. Frustules, due to the highly ordered distribution of pores in the thin silica cover layer, have the ability to waveguide light and act as natural biophotonic crystals [21]. A material modification is an important approach to change the physicochemical properties or functions of siliceous diatom shells and offers great opportunities for the synthesis of new materials with 3D nanostructures [22]. In this way, it is possible to obtain a material characterized by enhancing the unique optical properties of diatomaceous biosilica while the specific properties of the doped element are acquired [23]. Particularly promising are the already obtained research results concerning the possibility of doping diatomaceous biosilica with titanium to produce materials characterized by specific photo- and electroluminescent properties [5,24,25,26,27,28,29,30,31,32].

**Figure 1:**
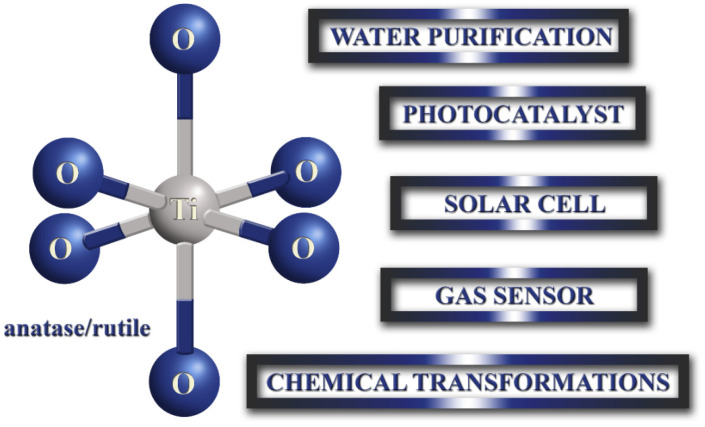
The range of possible applications of titanium dioxide in modern technologies.

In its dominant quantity in nature, titanium exists in the form of titanium oxide, in which it takes the +IV oxidation degree [33]. TiO_2_ is also the most stable titanium oxide [34]. Titanium dioxide exists in three different crystallographic forms: rutile, anatase (with tetragonal structure) and brookite (with orthorhombic structure), but only the rutile and anatase phases are of practical significance [35]. In both structures, the basic building block is a titanium atom surrounded by six oxygen atoms in a more or less distorted octahedral configuration [36]. TiO_2_ is also well known as a semiconducting oxide. It is inexpensive, chemically stable and harmless and it shows no absorption in the visible light region. In contrast, it absorbs UV light, generating a pair of electrons and holes which cause chemical reactions on its surface. For this reason, its most promising feature is its photochemical properties, such as high-photocatalytic activity [37]. Already since the 1950s, TiO_2_ has been extensively studied for its use as a photocatalyst [38,39,40], solar cell component [41] and gas sensor [42]. Additionally, titanium dioxide nanoparticles are used in the photocatalytic degradation of toxic chemicals in water [43,44] (Figure 1). However, the fabrication process of titanium nanoparticles requires high temperature, pressure and toxic chemicals, that limit their production and potential application [45]. Therefore, an environmentally friendly and cost-effective approach is needed to synthesize these nano-sized materials on a larger scale with fewer hazards.

A natural solution to this problem seems to be the modification with titanium oxides of a natural carrier that would be produced in an environmentally friendly way, at low cost and one that would exhibit biocompatibility [46]. The ideal “carrier” of this type seems to be diatoms with their adsorption and ability to incorporate atoms of elements other than silicon into their frustules [7]. According to previously published reports, diatomaceous biosilica has already been functionalized with TiO_2_ for use as an effective photocatalyst for indoor air purification [47,48], for the photocatalytic degradation of rhodamine B [29,49], as a catalyst for the photodegradation of methyl blue [50] and as a photocatalyst in the reduction in acetaldehyde [51].

A method for the metabolic insertion of titanium ions into diatom cells was first developed by C. Jeffryes et al. [31], using a two-step culture process of an unnamed species representing *Pinnularia*. The titanium precursor was a TiCl_4_ solution. Skolem [26] followed the same approach with a two-step process of doping siliceous frustules of diatoms *Pinnularia* sp. and *Coscinodiscus* sp. with titanium ions in a photobioreactor using the same titanium ion precursor as Jeffryes. Chauton and co-workers [24] also used a two-step process to dope *Pinnularia* sp. with titanium ions using the same precursor. They initiated titanium uptake when the silicon concentration in the culture medium dropped to below 0.5 μM. In the study by Eynde et al. [51], the two-step scheme of the *Pinnularia* sp. doping process was analogous, differing only in the timing of the addition of the titanium precursor, which occurred at the end of cell growth rather than afterwards. Studies on two-step doping of *Fistulifera solaris* by Maeda et al. [25] used titanium (IV) bis (ammonium lactate) dihydroxide (TiBALDH) as a precursor. Other research groups used a one-step doping process. In the work of Basharina [5], *Synedra acus* was cultured in microincubators in which 10 mM Na_2_SiO_3_ and 10 mM TiCl_4_ were added simultaneously to the alkali solution. A similar approach was used by Lang et al. [30], by adding 0.2–2.0 mM TiBALDH to the medium with *Thalassiosira weissflogii*.

The aim of this work was to develop a method for the biosynthesis of three-dimensional diatomaceous biosilica doped with titanium ions during the controlled culture of a selected species of diatom of the *Pseudostaurosira* species. The biological integration of TiO_2_ into silica cell walls has recently been demonstrated by simply culturing diatom cells in the presence of water-soluble Ti precursors (e.g., titanium oxysulfate, TiOSO_4_; titanium(IV) chloride, TiCl_4_ and titanium(IV) bis(ammonium lactate)dihydroxide, TiBALDH) [24,30,31]. These approaches indicate that the highest percentage of titanium incorporation was achieved with TiBALDH, which is an organic titanium precursor. For this reason, in this work, another organic precursor of titanium and titanium terbutoxide was used. The specific objectives of the work included the study of morphological and structural features, elemental composition, photoluminescence properties and thermal stability, using a range of instrumental methods including scanning electron microscopy, transmission electron microscopy, UV/Vis photoluminescence spectroscopy, FTIR spectroscopy, X-ray powder diffraction, thermogravimetry and zeta potential.

## 2. Materials and Methods

### 2.1. Diatom Cultivation Process

A clonal strain of the diatom species *Pseudostaurosira trainorii* was obtained from the Baltic Algae Culture Collection of the Institute of Oceanography, University of Gdansk, Gdansk, Poland. The diatoms were cultured in 25 L photobioreactors containing Guillard’s medium F/2 [52]. A source of soluble silicon in the form of sodium metasilicate (Na_2_SiO_3_ × 5H_2_O) with a concentration of 7–300 mg/L and a source of soluble titanium with a concentration ranging from 2.5 to 90 mg/L were added to the culture medium. The soluble titanium source was obtained by dissolving titanium(IV) tertbutanolate (C_16_H_36_O_4_Ti) in 37% hydrochloric acid. The pH of the culture medium was adjusted to 8.9 with 0.1 M NaOH. The culture was grown at room temperature (approximately 25 °C) under constant aeration and 1500 lux illumination at day/night intervals of 12:12 h for 12 days. After the growing period, the biomass obtained was filtered through Macherey Nagel 616 G filters (Düren, Germany) using a vacuum pump, washed with distilled water and dried at 70 °C.

### 2.2. Purification of Diatom Frustules

Diatomaceous biosilica doped with titanium ions was isolated from dried biomass using a 30% H_2_O_2_ solution to remove organic matter from diatom cells. The process was carried out at 80 °C for 4 h. At the end of the process, a few drops of 37% hydrochloric acid were added to remove calcium carbonates and residual H_2_O_2_. The obtained diatomaceous biosilica was washed with distilled water and left to sediment for 24 h. Then the solution was decanted from the pure biosilica. The resulting sediment was centrifuged on an Eppendorf Centrifuge 5810 R (Hamburg, Germany) at 10,000 rpm for 10 min. Subsequently, the pure biosilica was washed and centrifuged again. The washing and centrifugation process was repeated five times. Finally, the obtained diatomaceous biosilica doped with titanium ions was left to dry at 120 °C.

### 2.3. Instrumental Methods

The growth rate of diatom cells was controlled by daily measurement of diatom optical biomass density using a BIOSAN DEN-1B densitometer (Riga, Latvia) at 565 nm.

The uptake by diatom cells of elements associated with diatom cell formation and growth were monitored photometrically using Spectroquant^®^ tests. The tests performed were compatible with the Spectroquant NOVA 60 spectrophotometer and concerned the uptake of silicon (Merck Spectroquant^®^ test kit no. 1.14794, Kenilworth, NJ, USA), phosphorus (Merck Spectroquant^®^ test kit no. 1.14848) and nitrogen (Merck Spectroquant^®^ test kit no. 1.09713).

Titanium uptake by diatom cells was monitored using a Shimadzu ICP-MS 2030 (Kyoto, Japan) inductively coupled plasma mass spectrometer. Samples of 5 mL containing the culture medium were collected daily for 12 days of culture. The collected samples were then diluted 100-fold in ultrapure 1% HNO_3_.

To analyze the surface morphology of the obtained material, a Quanta 3D FEG scanning electron microscope SEM/FIB with a resolution capacity of 1.2 nm and SE signal detection was used (Boynton Beach, FL, USA). Before analysis, samples were sputtered with a nanometric gold layer. The analysis was carried out in variable vacuum mode. The elemental composition and distribution of titanium were studied by SEM-EDX.

Morphological features, pore structure and the elemental composition of diatom frustules and the biomass doped with titanium ions were analyzed by transmission electron microscopy (TEM) and scanning transmission electron microscopy (STEM-EDS) using a TEM (FEITecnai F20 X-Twintool, Lincoln, NE, USA) coupled to an Energy Dispersive X-ray (EDX) detector with sample placement on a carbon-coated copper grid (Lacey Carbon Support Film 400 mesh).

X-ray powder diffraction analyses (XRD, Malvern Panalytical Ltd., Malvern, UK) were performed using a Philips X’Pert Pro with Cu-Kα radiation (λ = 0.1541 nm, 40 kV, 30 mA). Titanium ion doped diatomaceous biosilica samples were scanned over an angular range of 5°–120° 2θ with a step of 0.01.

The zeta potential values of biosilica and biomass doped with titanium ions were determined using a Zetasizer Nano Series instrument (Malvern Instruments). Prior to measurement, the analyzed samples (at a concentration of 0.25 mg/mL) were suspended in water at a specified pH (range 1 to 12) and sonicated in a Polsonic ultrasonic bath for 360 min at 25 °C. Measurements included three replicates for each sample.

The thermal stability of the obtained materials was tested in the temperature range 20–1000 °C using a Jupiter STA 449 F5 thermoanalyzer from Netzsch (Selb, Germany). The samples were heated at a heating rate of 10 °C min^−1^ in a nitrogen atmosphere. After completion of the analysis, the samples were used as pyrolysis samples and examined by XRD.

Structural bonds and functional groups were recorded using a Fourier Transform Infrared (FTIR) spectrophotometer (FTIR ATR, Vertex 70, Bruker Optik, Billerica, MA, USA) equipped with a DLaTGS detector and a Senterra dispersive Raman spectrometer (Bruker Optik). FTIR spectra were recorded by averaging 64 scans in the wave number range from 400 cm^−1^ to 4000 cm^−1^ with a resolution of 4 cm^−1^.

The photoluminescence (PL) properties were investigated using an FP-8300 spectrofluorometer (FP-6500, JASCO, Madrid, Spain) equipped with a xenon lamp. PL spectra were measured at excitation wavelengths in the UV-VIS range from 200 to 750 nm. Measurements were performed at room temperature using a colloid solution of the obtained material at a concentration of 0.014 mg biomass or biosilica per 1 mL in water and a quartz cuvette of 1.0 cm diameter with correction of the primary spectra.

## 3. Results and Discussion

### 3.1. Equilibrium Study of the Titanium Uptake by Diatom Cells. Effect of pH Value, Initial Ti Concentration and Ti:Si Ratio in the Culture Medium

The equilibrium study (values testing on the last day of cultivation) of titanium incorporation into the diatom cells, as well as cell density growth of the diatom species *Pseudostaurosira trainorii,* was carried out in four separate series of experiments. The initial experiment (Series I) consisted in the selection of an appropriate pH value, being a compromise between the optimum growth of the diatoms and the amount of incorporation of titanium into the diatom frustules. The second series (Series II) focused on the selection of the optimal initial concentration of titanium ions, added to the culture medium, in order to obtain the best possible biomass production yield and optimal amount of incorporated titanium. The third and fourth series (Series III, Series IV) consisted of determining the appropriate mass ratio of the initial concentrations of dissolved titanium and silicon in the medium, to obtain the optimum amount of material containing the highest weight percentage of incorporated titanium.

**Figure 2:**
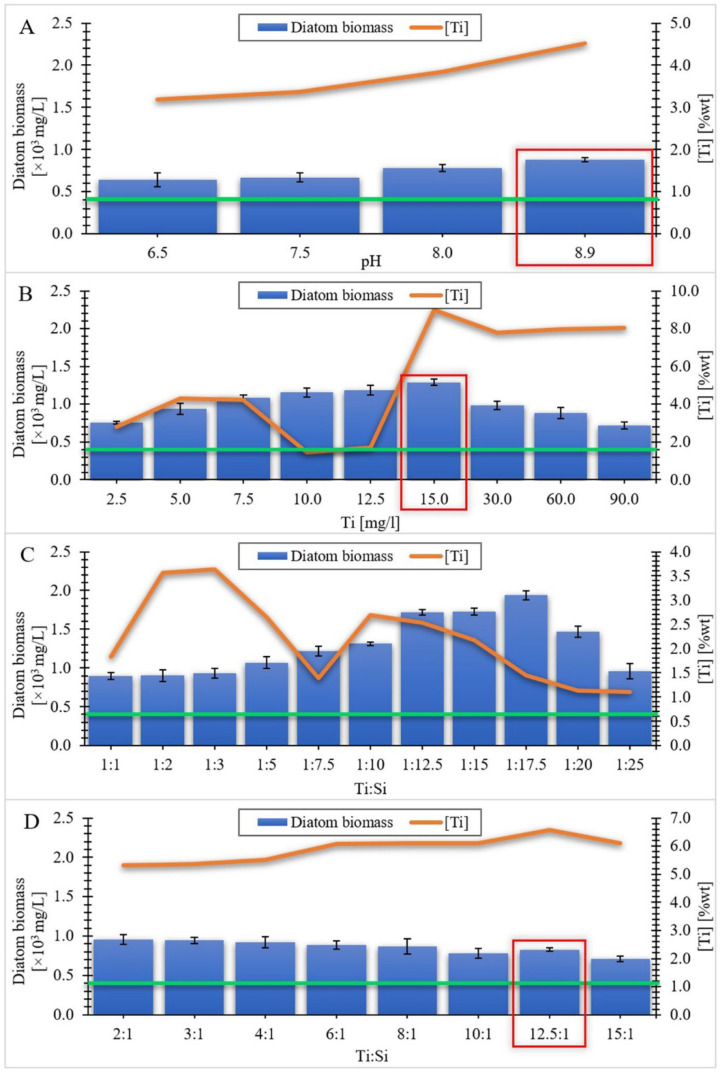
The amount of biomass per last day of culture as a function of (**A**) pH value; (**B**) Ti concentration; (**C**,**D**) Ti:Si ratio in relation to titanium content in diatom biomass in % weight (orange line). The undoped cell production value from the diatom species *Pseudostaurosira trainorii* is indicated by the green line. The red rectangle indicates the key samples for a given series of experiments.

In this study we used McFarland measurements of undoped diatom biomass to standardize measurements of the kinetics of doped materials. We also quantified diatom cells without titanium doping in a haemocytometer and determined that a suspension with a turbidity of 1 McFarland unit is approximately equivalent to 4 × 10^5^ cells/mL [53]. The production efficiency of undoped cells from the species *Pseudostaurosira trainorii* was approximately 0.39 mg/mL. This value is presented as a green line in Figure 2.

Figure 2 shows histograms illustrating the amount of biomass obtained [in mg/L] on the last day of cultivation for each series. The general conditions of controlled cultivation of diatoms doped with titanium ions for all series carried out are summarized in Table 1.

#### 3.1.1. Series I

The tests were carried out for four pH values which were 6.5, 7.5, 8.5 and 8.9 respectively. The selection of the pH range for the experiment was motivated by the living pH range for the species *Pseudostaurosira trainorii*. Furthermore, as the pH increases, the possibility of destroying the siliceous shell of the diatoms increases [54], which would have been an undesirable effect, as the intention was to preserve the intact, three-dimensional structure of the diatom frustule. The initial concentration of soluble silicon was 7.0 mg/L, whereas the initial concentration of soluble titanium was 2.5 mg/L. The results of the experiments carried out related to Series I are shown in Figure 2A. It was observed that the amount of obtained biomass increased with increasing pH. After 12 days, the highest amount was recorded in the flask containing culture medium with doped titanium at pH = 8.9. The obtained results are confirmed by the literature data [55], in which the optimal pH values for the cultivation of microalgae were estimated in the range of 8.2–8.9. It is also visible that the amount of incorporated titanium in diatom cells also increases with the increase in the pH value (Figure 2A). This may be related to the fact that in the basic pH range, titanium is present mainly as Ti(OH)_4_, and silicon is absorbed by diatoms as Si(OH)_4_, hence titanium in this form can be absorbed into the cell in the same way as silicon [56]. Regardless of the pH of the culture medium used, the biomass value on the last day of cultivation was higher than the reference value of the amount of biomass of undoped diatom cells (it applies to all the series). In the works related to the metabolic insertion of titanium ions into diatom cells, a significant increase in biomass was observed during the experiment [24,30]. Only in the experiment carried out by Skolem [26], the efficiency of diatom biomass was lower compared to the blank test.

#### 3.1.2. Series II

In this series of experiments, tests were performed for nine initial values of soluble titanium concentration in the culture medium, which were 2.5, 5.0, 7.5, 10.0, 12.5, 15.0, 30.0, 60.0 and 90.0 mg/L, respectively. The initial concentration of soluble silicon was 7.0 mg/L and the pH of the culture solutions was maintained at 8.9. The selection of the pH value was the result of the conducted experiments in Series I. The results of the tests concerning series II are shown in Figure 2B. When analyzing the obtained values, it can be noticed that up to a certain concentration of titanium, known as the limit concentration, the amount of obtained biomass increases with the increase in concentration. After exceeding the limit concentration, the mass of diatom cells incorporated with titanium decreased. This behavior may be caused by exceeding the tolerance threshold of the stressor, which are titanium ions for diatom cells [57]. In addition, the research conducted by Eynde et al. [47] on *Pinnularia* sp. cultures showed that the inhibition of the cell growth process may also depend on the type of titanium precursor used in the culture medium. Considering the obtained results of the content of incorporated titanium depends on the increasing concentration of this element in the culture medium, there is no visible relationship between the amount of biomass and the amount of absorbed titanium. The highest incorporation value of the doped element (8.98% wt) was obtained for the material in which the concentration of soluble titanium in the culture medium was 15 mg/L.

#### 3.1.3. Series III

This series consisted of eleven tests, in which the mass ratios of Ti:Si were respectively: 1:1, 1:2, 1:3, 1:5, 1:7.5, 1:10, 1:12.5, 1:15, 1:17.5, 1:20 and 1:25. The initial concentration of soluble titanium was 15 mg/L (which was a consequence of the results of the experiments in Series II) and it was constant for all experiments. The concentration of silicon in the culture medium varied depending on the experiment and ranged from 15 mg/L for the first test (1:1) to 375 mg/L for the 1:25 test. A graphical presentation of the series III results is shown in Figure 2C. It is visible that, as in the case of increasing the concentration of titanium in series II, also in this series, the increase in silicon concentration in the culture medium causes an increase in the amount of obtained biomass. This increase occurs until the limit concentration is reached (with ratio Ti:Si = 1:17.5), beyond which a decrease in the obtained diatom cell mass is observed. This may be due to the fact that Si incorporation is mainly related to the duration of the synthesis of the cell wall. Due to the fast growth rate of the *Pseudostaurosira* strain, exceeding the limit concentration of silicon causes a reduction in the incorporation of silicon into the frustules, and thus a decrease in the number of cells [58,59]. The amount of biomass (1.94 × 10^3^ mg/L) at the point corresponding to the critical concentration was the highest compared to the amount of biomass obtained in the other series. When analyzing the results of the value of incorporated titanium, it can be noticed that there is no visible correlation between the increase in silicon concentration and the increase in titanium uptake into diatom cells. However, it is evident that the obtained values of the titanium content in the doped biomass are lower than in the case of Series II, where the initial Si concentration was 7 mg/L. This is probably due to the insufficient concentration of titanium in the culture medium in relation to the amount of silicon contained in it.

#### 3.1.4. Series IV

The tests were performed for eight values of the Ti:Si mass ratios, in which titanium was the dominant element. The values of the above ratios were respectively: 2:1, 3:1, 4:1, 6:1, 8:1, 10:1, 12.5:1 and 15:1. The initiating concentration of soluble titanium in the culture medium was 15 mg/L (which was a consequence of the results of the experiments in Series II) in all experiments in this series. The silicon content in the culture medium varied depending on the experiment and ranged from 1 mg/L for the last test (15:1) to 7.5 mg/L for the 2:1 test. The results of the experiments related to series IV are presented in Figure 2D. It can be noticed that the amount of obtained biomass decreases with the decrease in the silicon concentration in the culture medium. This result is in line with the results obtained in Series III. This may be due to the fact that the amount of silicon supplied to the medium is not sufficient to cover the demand of diatom cells for this element in the cycle of cell wall formation, due to the rapid growth rate of this species [60]. The observed decrease in the amount of obtained biomass is visible until the initial silicon concentration in the culture medium is 1.5 mg/L. When the value of the silicon concentration drops to 1.2 mg/L, a slight increase in the biomass of diatoms is visible. When the value drops to 1 mg/L, another decrease in the amount of biomass occurs. Considering the obtained results of the content of incorporated titanium depend on the decreasing concentration of silicon in the culture medium, it is visible that with the decrease in the amount of silicon in the medium, the amount of titanium absorbed by the cells increases. This is the case up to the point where the silicon concentration in the medium is 1.2 mg/L. This is the point for which the titanium content in diatom cells is the highest, amounting to 6.57%. After exceeding this value and reducing the silicon content in the medium to 1 mg/L, a slight decrease in the amount of incorporated titanium into diatom cells is visible.

After carrying out the above series of experiments related to the selection of the appropriate pH, titanium concentration and the appropriate ratio of titanium to silicon concentration, a representative material was selected, which turned out to be a compromise between the amount of biomass obtained and its titanium content. This material turned out to be biomass doped with titanium ions, with an initial concentration of 15 mg/L (and the initial silicon concentration of 1.2 mg/L), subject to further analyses, the results of which are described in this article. In the further part of the work, the biomass doped with titanium ions with a concentration of 15 mg/L will be designated as Ti_15_g, whereas the doped biosilica will be referred to as Ti_15_w. Reference materials such as undoped biomass and pure diatomaceous biosilica will be expressed with the symbols N_g and N_w respectively.

### 3.2. Kinetics of the Components (Si, N, P and Ti) Uptake by Diatom Cells from the Culture Medium

**Figure 3:**
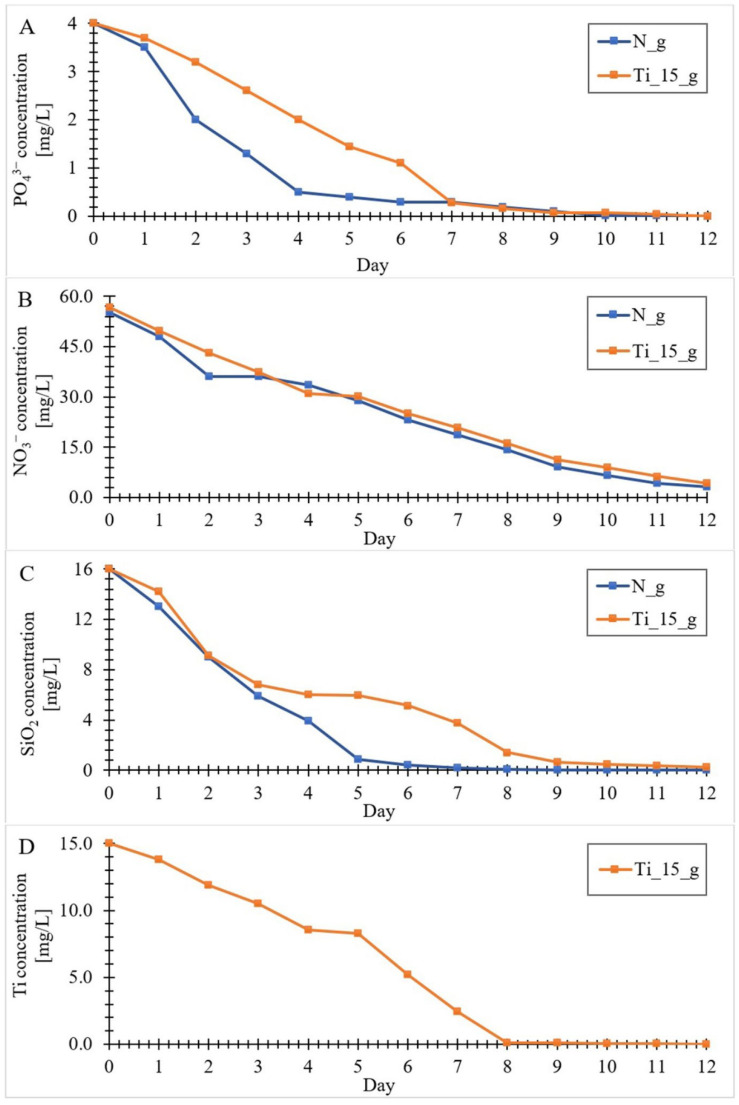
Kinetics of the components’: P (**A**), N (**B**), Si (**C**) and Ti (**D**) uptake by diatom cells from the culture medium. (Ti_15_g-the biomass doped with titanium ions with an initial concentration of 15 mg Ti/L; N_g-undoped biomass).

The rates of nitrates, phosphates and silicon into diatom cells’ uptake (undoped and in the presence of titanium ions with the initial concentration of 15 mg/L) was estimated during 12-day experiments using photometric tests. In addition, throughout the entire culture cycle, analyses of the culture medium samples were carried out using ICP-MS to show the uptake of titanium ions by diatom cells. The obtained results are shown in Figure 3. As can be seen in the attached graphs, the largest part of these elements is absorbed in the first few days. The obtained results are confirmed by literature studies related to the growth kinetics of microalgae, which indicate that during the first few days there is a phase of intensive growth of diatom cells [30,61,62,63,64]. During this period, the nutrients do not limit the growth of the microalgae. In the growth phase, the division of microalgae cells slows down, causing the biomass of the microalgae to accumulate at a constant rate until the ingredients in the culture medium become limiting factors [65]. They then reach a stationary phase that coincides with the depletion of phosphorus and silicon in the culture medium. In our study, the concentration of PO_4_^3−^ decreased to <0.008 mg/L (Figure 3A). Additionally, the concentration of SiO_2_ decreased significantly, reaching <0.011 mg/L (Figure 3C), whereas NO_3_^−^ decreased practically linearly, without becoming a limiting element. (Figure 3B). The fact that NO_3_^−^ did not become a limiting factor may indicate that it is not the main source of N in the medium. The source of nitrogen production in the culture medium can be organic nitrogen, derived from the excretion of microalgae occurring during death or lysis, as well as in the phase of productive growth [66]. Due to the high concentration of the obtained biomass and the fast growth and division rate, one could expect a high concentration of organic nitrogen in the medium [67,68], but such studies were not the subject of this study. Analyzing the titanium ion absorption curve by diatoms (Figure 3D), it can be noticed that its shape is similar to the shape of the silicon absorption curve. As in the case of soluble silicon, the greatest uptake of titanium to diatom cells occurred in the first few days of culture. It was observed that on the eighth day the amount of soluble titanium in the culture medium dropped to <0.1 mg/L. Due to this decrease, titanium ions became a limiting element, which resulted in an inhibition of titanium uptake into diatom cells. Considering that the absorption of titanium from the culture medium by biomass was similar to the absorption of silicon (as shown by the Figure 3C,D curves), it can be concluded that the scheme of titanium and silicon diatoms uptake into cells is the same [56], and the absorption of these elements takes place in parallel.

### 3.3. Scanning Electron Microscopy Analysis

**Figure 4:**
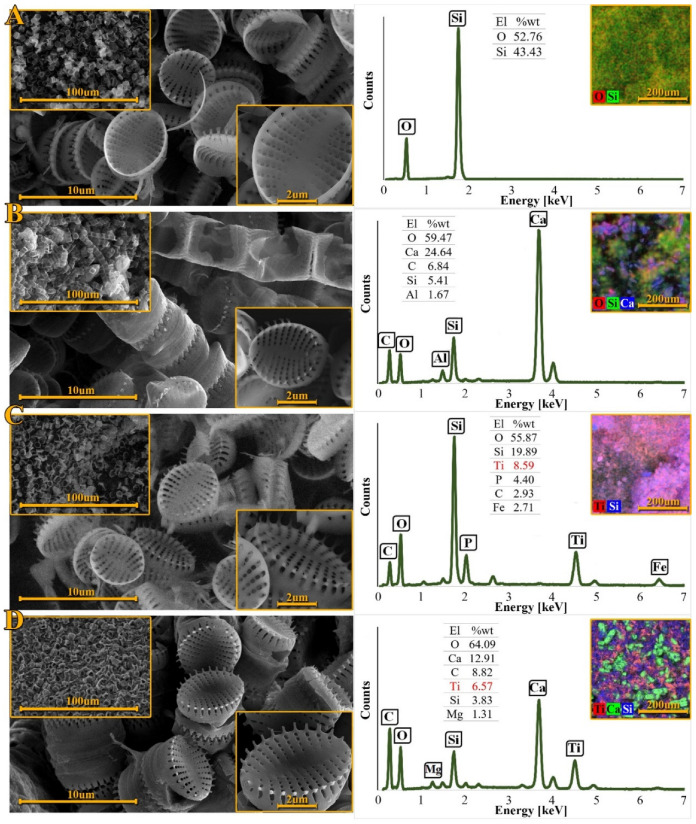
Review of SEM images and SEM-EDX spectra, elemental composition and distribution of titanium: (**A**) purified diatomaceous biosilica, (**B**) dried biomass, (**C**) diatomaceous biosilica doped with titanium ions, (**D**) diatomaceous biomass containing titanium.

Figure 4 shows an overview of SEM pictures of the materials obtained. Figure 4A shows purified and dried undoped diatomaceous biosilica. It is visible that the intricately ornamented, three-dimensional structure of the diatom shells has been preserved, along with its entire, periodically distributed network of pores. Individual diatom shells are elliptical or flattened spheroids in shape and their diameter ranges from 4 to 5 μm [69]. A strongly silicified sternum runs in the center of the valves and divides it into two halves with eight to twelve transapical striae. The striae consist of about 3–5 pores, the size of which decreases from the valve margin to the center [53]. The SEM-EDX analysis confirmed that the diatom frustules consist of oxygen and silicon, which proves that the purification process resulted in the complete removal of the diatom cell’s organics, i.e., protoplasm and chloroplasts [70].

Figure 4B shows the filtered and dried biomass without the doped element. It can be seen that the diatom cells of the species *Pseudostaurosira trainori* from the perspective of the girdle have a rectangular shape and form ribbon-like colonies connected by spines [60]. The elemental composition of diatom biomass was obtained using SEM-EDX. Additionally, in order to complete the elemental analysis of the obtained material, XPS tests can be performed, as evidenced by the works of Uthappa et al. [71] and Aw et al. [72].

These data showed that the main components of diatom biomass were oxygen, carbon, silicon and calcium. The presence of calcium may indicate the formation of calcium carbonates, which are visible on the EDX mapping as green forms (Figure 4D). The obtained forms are similar to the forms of calcium carbonates obtained in the study of El-Naas et al. [73]. Additionally, in our previous work, the presence of calcium carbonates in the biomass of *Pseudostaurosira trainorii* diatoms was confirmed by XRD, TG and FTIR analysis [74]. The significant carbon content in the sample indicates the presence of the organic part of the diatom cell. Figure 4C shows the purified and dried titanium ion doped diatomaceous biosilica, whereas Figure 4D shows the filtered and dried titanium doped biomass. It is visible that under the influence of titanium ions the oval shape of the diatom cells remained intact.

Regular arrangements of the pores create periodic patterns that make up the frustule ornamentation [28]. The obtained materials show a morphology similar to that of the undoped diatom frustules. Figure 4D shows that the surfaces of the diatom cells are partially rough. This may be due to the organic substances overlaying them which form the organic cell structures [29]. After the purification process, the organic substances were removed and the siliceous diatom shells were exposed, as shown in Figure 4C. The surface of the purified diatom frustules is smoother, which indicates that the process of removing organic matter from diatom cells has been successful. SEM-EDX analysis also showed the presence of titanium in both samples. Mapping indicates an even distribution of the doped element in the materials. The mass percentage of titanium in the purified biosilica was higher (8.59%) than in the biomass (6.57%). The presence of Fe and P in the purified biosilica may be the result of the fact that the purification process did not completely remove the residues from the culture medium.

### 3.4. Transmission Electron Microscopy Analysis

**Figure 5:**
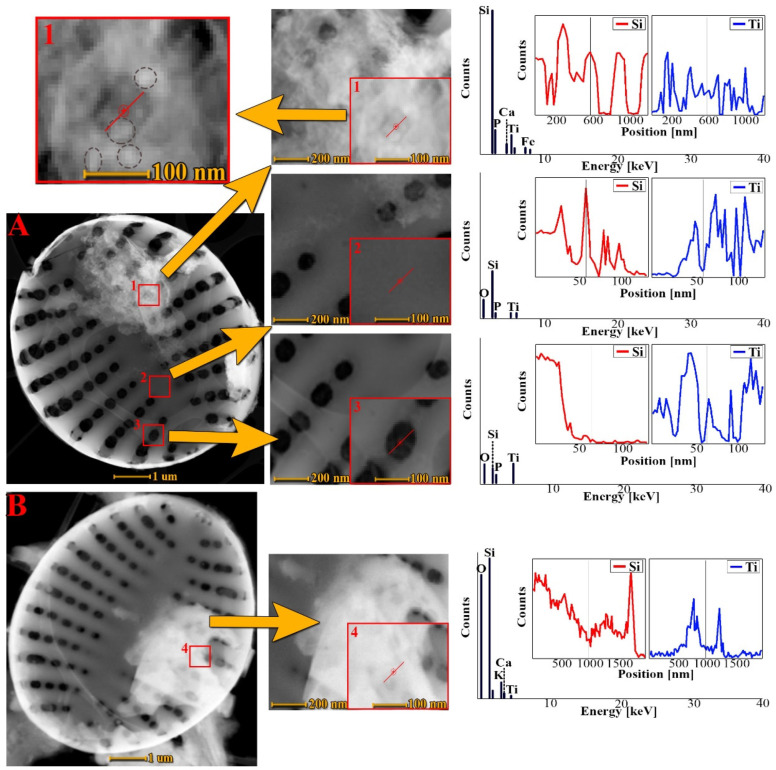
TEM images of diatomaceous biosilica (**A**) and biomass (**B**) doped with titanium ions (**A**), STEM-EDX spectra and scans of elementary lines of Ti and Si in four representative places of diatom cells: on the surface of a flake of purified biosilica (A.1.), on ribs (A.2.) in the pore cross-section (A.3) and on the surface of the flake incorporated on the cell of the unpurified biomass (B.4). In the zoom of figure A.1, the dashed black line shows titanium quasi-ooid forms.

Figure 5 shows the comparison of transmission electron microscopy images of purified biosilica with metabolically inserted titanium (Figure 5A), with biomass doped with titanium ions (Figure 5B). TEM images show in detail the intricate structure of diatomaceous frustules. The architecture of the entire shell of diatoms is visible, as well as the details of the structure of individual pores. It can be noticed that the influence of titanium on the morphology of diatom cells is insignificant. As in the work of Basharina [5], the joining of some single pores into larger pores was observed; however, the overall structure of individual pores has not been changed. Jeffryes et al. [56] obtained a similar result in studies on the metabolic incorporation of germanium into diatomaceous biosilica. However, this phenomenon was more intense than in our case. No such changes were observed during the doping of diatom cells of the *Pinnularia* sp. species with titanium ions [31]. In his work [51], Van Eynde noticed that the increased amount of titanium in the hierarchical structure of diatom shells causes the effects of deformation and warping, causing irregularities in the structure [5,51,75].

On the surface of the frustule there are fragments of thin coat in the form of corrugated flakes of various sizes. The structure of these flakes is amorphous and irregular, resembling fluff. It also contains embedded, spherical forms of nanoparticles (ooids) with dimensions of 10–20 nm. The locations and sizes of these flakes vary for each individual frustule. However, their presence is characteristic of all the frustules of purified biosilica and cells of crude biomass doped with titanium ions. The thickness of the above-mentioned coatings does likely not exceed 20 nm. The size of the flakes ranges from 200 nm to 2 µm. Linear scans A.1. and B.1. indicate that the thin flake observed on the surface of the frustule contains specimens such as calcium and titanium, and the presence of Si and O elements confirms that the flake is directly bonded to the surface of the silica diatom shell, despite being etched with a solution of H_2_O_2_ and HCl. So far, no reports of the formation of similar coatings have been reported in the scientific literature. The nature of the binding phenomenon between the resulting flake and the siliceous surface of the diatom frustules remains unexplained. This bonding is probably due to the joining of silanol groups with the titanium flake composite. STEM-EDX analyses and STEM-EDX linear scans were performed in three representative places of the diatom frustules: on the surface (Figure 5 A.1), on the rib (Figure 5 A.2), inside the pore (Figure 5 A.3) and in one place of the cell diatoms—on the surface of the “flake” (Figure 5 B.4).

Linear scans on Figure 5 A.2 and Figure 5 A.3 show that Si, Ti and O were the main elements present in the frustule. Linear STEM-EDX scans for titanium and silicon indicated that the Ti signals were also detectable in the flake-free sites. This may suggest that the silicon in the biosilica has likely been partially metabolically substituted by titanium. This could be possible due to the fact that titanium and silicon have similar covalent radii [51,75]. Additionally, linear scans indicated that the highest Ti counts were concentrated on the surface of the flakes, then inside the pore, and the smallest in the shell structure (in the rib). However, it should be emphasized that the number of signal counts is approximate, because the EDS measurement depends on the penetration and scattering of the X-ray beam in the sample [76] and the surface roughness [77].

### 3.5. X-ray Powder Diffraction Patterns

**Figure 6:**
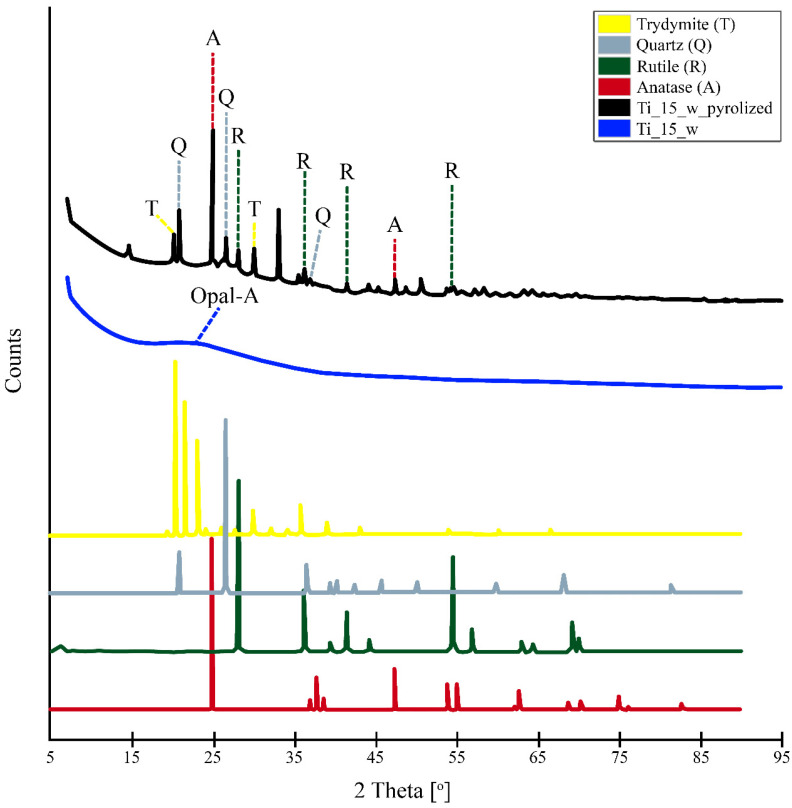
X-ray powder diffractograms of dried diatomaceous biosilica doped with titanium ions and diatomaceous Ti-biosilica subjected to pyrolysis at 1000 °C. XRD patterns for the patterns have been retrieved from the ICDD database and are characterized by the following numbers: anatase: JCPDS 21–1272, rutile: JCPDS 21–1276, tridymite: JCPDS 18–1170 and quartz: JCPDS 46–1045. (Ti_15_w-the biosilica doped with titanium ions with a concentration of 15 mg Ti/L; Ti_w_pyrolized-the biosilica doped with titanium ions with a concentration of 15 mg Ti/L pyrolized at 1000 °C).

X-ray powder diffraction patterns of diatomaceous biosilica doped with titanium ions, dried at 50 °C, and diatomaceous Ti-biosilica, pyrolized at 1000 °C, are shown in Figure 6. The XRD spectrum obtained with dried titanium-containing diatomaceous biosilica showed a broad peak at 15–30° 2θ, which can be attributed to amorphous silica, such as opal-A [78,79]. In the above range of angles there is also 25° 2θ, which is characteristic of the crystalline phase of TiO_2_ in the form of anatase. The lack of visibility of the above-mentioned crystal phase may be due to the low content of titanium in the biosilica compared to the large matrix of amorphous silica, which makes it difficult to accurately detect the crystalline phase and makes the sample Ti_15_w without pyrolysis is amorphous [51]. The temperature increase to 1000 °C caused the transition from the amorphous phase of the titanium-doped biosilica to its crystalline phases. Two crystalline forms of TiO_2_ (such as anatase and rutile) and SiO_2_ (such as quartz and tridymite) have been observed. The XRD spectrum obtained for the titanium-containing diatomaceous biosilica, annealed at 1000 °C, showed diffraction peaks at 25.22 and 47.85, which are consistent with the peaks of the anatase crystalline form. The diffraction peaks at 27.39, 35.97, 41.11 and 54.12 correspond with the peaks’ characteristic of rutile TiO_2_.

The appearance of the rutile crystalline phase is conditioned by the irreversible phase transition from the anatase form, which occurs at temperatures above 600 °C [80]. Earlier studies indicate that the precipitation of TiO_2_ or TiP_2_O_7_ from soluble precursors with peptides or proteins results in the amorphous forms of the above-mentioned compounds. However, thermal annealing may result in a phase transition from amorphous to crystalline [81,82,83]. In several studies using the biomimetic approach, crystalline forms of TiO_2_ were obtained without thermal annealing.

Kröger et al. [84] observed that the precipitation of titanium dioxide from the TiBALDH precursor by the rSilC recombinant silafin protein resulted in the formation of rutile TiO_2_. Dicerson et al. [85] observed that 12-mer phase-display peptides produce a mixture of amorphous, anatase, and monoclinic (β) forms of TiO_2_. Bansal et al. [86] used the titanium precursor K_2_TiF_6_ to precipitate, at room temperature, the brookite form of TiO_2_ with the help of proteins secreted from the fungus *Fusarium oxysporum*. Jeffryes et al. [31] by thermal annealing of the material obtained during the metabolic insertion of titanium ions into the diatomaceous biosilica of the *Pinnularia* species, obtained a mixture of two types of TiO_2_ crystal forms: anatase and rutile. In the XRD spectrum of Ti_15_w_pyrolised, diffraction peaks at 20.87, 26.64 and 36.54 were also observed, which were consistent with the peaks’ characteristic for quartz. Diffraction peaks at 20.47 and 29.96, which correspond with the peaks’ characteristic for the SiO_2_ crystal form, which is tridymite, were also noticed. It is now well known that α-quartz is stable at about 570 °C. Above this temperature, it transforms into β-quartz through at least one disproportionate intermediate phase in the temperature range from 573 to 574.1 °C [87]. At a temperature of approx. 870 °C, there is a phase transition from β-quartz to β_2_-tridymite, which is highly stable up to 1470 °C.

### 3.6. Zeta Potential Measurements

**Figure 7:**
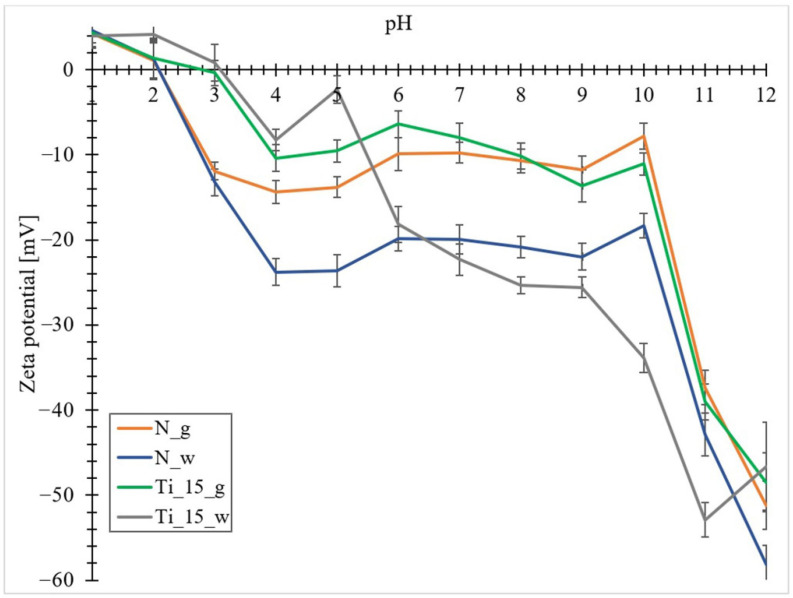
The zeta potential performed in the pH range with values from 1-12, for the analyzed materials (N_g-undoped biomass; N_w-pure diatomaceous biosilica; Ti_15_g-the biomass doped with titanium ions with a concentration of 15 mg Ti/L; Ti_15_w-the biosilica doped with titanium ions with a concentration of 15 mg Ti/L).

The stability of the obtained biomaterials was estimated by measuring the zeta potential. The zeta potential (ZP) is defined as the electrokinetic potential occurring at the boundary between the bound and free liquid, between the ions bound immobile in the adsorption layer and the free counter ions of the diffusion layer, named the shear plane. Consequently, the zeta potential is proportional to the charge density on the colloid surface, which depends on pH [88]. The dependence of the zeta potential on pH for the materials obtained is shown in Figure 7. It is noticeable that for biomaterials not doped with titanium, the zeta potential shows positive values in the pH range from 1–2 (ζ-potential from +1.1 mV to +4.6 mV) and negative values from 2 to 12 (ζ-potential from −9.8 mV to −58.1 mV).

The zeta potential of materials doped with titanium takes positive values in the pH range from 1 to 3 (ζ-potential from +0.8 mV to +4.3 mV) and negative values from 3 to 12 (ζ-potential from −0.4 mV to −52.9 mV). The sharp decrease in zeta potential in the pH range from 1 to 4 can be attributed to the ionization of carboxyl groups [89], particularly for materials obtained from diatom biomass.

The occurrence of a negative zeta potential value in such a wide pH range can be attributed to the abundance of silanol groups on the surface of frustules [90], as well as to the adsorption of cations present in seawater (e.g., Ca^2+^, Mg^2+^) on the surfaces of frustules, which cause more nano-TiO_2_ particles to be adsorbed on the surfaces of diatom shells [91].

According to literature data, most microorganisms show a negative zeta potential. For example, green alga *Chlorella* sp. had a relatively low ZP between −17.4 and 19.8 mV regardless of pH (4–10) [89] and the ZP of *Nitzschia* sp. diatom was −28 mV in stationary phase [92]. The apparent isoelectric point (IEP) around pH = 2 for materials undoped with titanium, which coincides with the isoelectric point of amorphous silica SiO_2_ [93].

The shift of the isoelectric point from pH = 2 to pH = 3 in doped materials is due to the presence of titanium in the analyzed samples. According to the literature, the isoelectric point of TiO_2_ nanoparticles suspended in distilled water is located around pH = 7 [94]. Liao and co-workers studied the effect of different TiO_2_ precursors on the zeta potential value as a function of pH. Using titanium(IV) terbutanolate, they observed a shift of the isoelectric point in the region of pH = 3 [95]. The lower isoelectric point suggests better stability of the particle dispersion around neutral pH due to the stronger repulsive electrostatic interaction. Furthermore, the lower IEP of TiO_2_ nanoparticles can be attributed to the presence of carbonates on the surface of TiO_2_ nanoparticles, as can be evidenced by the carbon signal in the EDX results (Figure 4). Kosmulski et al. [96] concluded that the presence of carbonate shifts the IEP of metal oxides to a lower pH. The obtained composites show the highest stability at pH 11 and 12, as for these pH values, each biomaterial has a zeta potential lower than −30 mV, which is the limiting potential for the stability of suspensions [97].

### 3.7. Thermogravimetric Analysis

**Figure 8:**
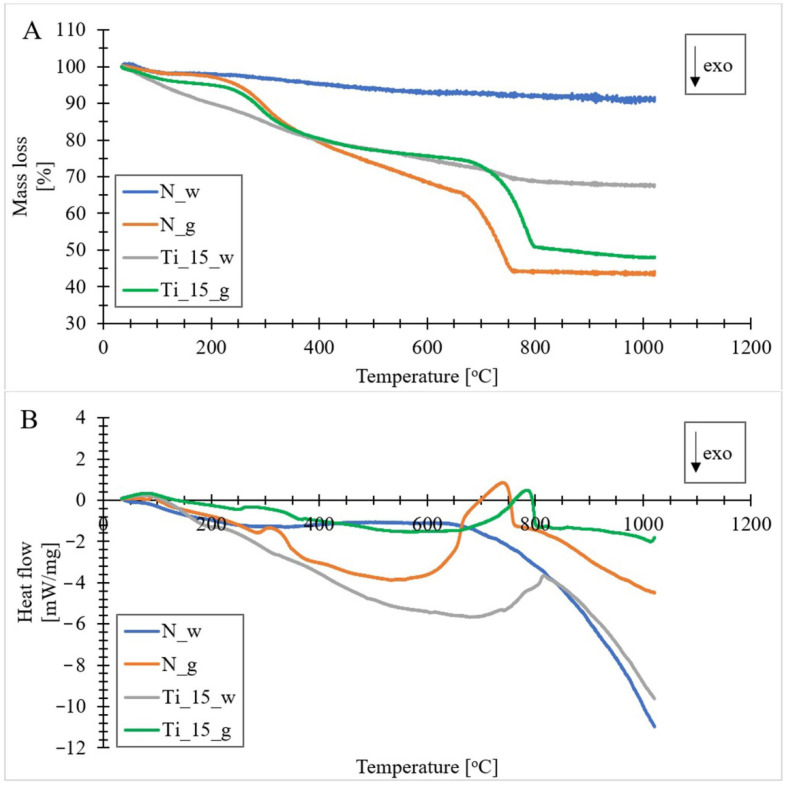
Graphs obtained from thermogravimetric analysis (**A**) and differential scanning calorimetry (**B**) of the obtained materials (N_g-undoped biomass; N_w-pure diatomaceous biosilica; Ti_15_g-the biomass doped with titanium ions with a concentration of 15 mg Ti/L; Ti_15_w-the biosilica doped with titanium ions with a concentration of 15 mg Ti/L).

Thermogravimetric (TG) analysis was carried out in a nitrogen atmosphere to investigate the thermal stability of the obtained materials (Figure 8A). In addition, during the TG analysis, the dependence of heat flow on temperature was measured by differential scanning calorimetry (DSC) (Figure 8B). In the process of controlled heating [98] of the analyzed samples, we can distinguish five stages of mass loss. The first phase is manifested on the TG curve in the temperature range from 40 to about 125 °C. The mass loss in this phase is different depending on the sample analyzed. For the sample containing undoped biosilica, the mass loss was about 3%. Samples of doped and undoped biomass showed a mass loss of about 5%, whereas the sample containing diatomaceous biosilica doped with titanium ions was characterized by a mass loss in the region of 11%. The mass loss in this phase may correspond with dehydration processes (release of physically loosely bound water) of the obtained materials [53]. The dehydration process is also confirmed by a small endothermic peak on the DSC curves. The second phase appeared in the temperature range from about 265 to about 360 °C, with a mass reduction of about 2% for sample N_w, and a value in the range of about 7–13% for the other samples. This is accompanied by an endothermic effect on the DSC curves. This phase could be related to the first step of the dehydroxylation process. It could be associated with the desorption of vicinal hydroxyl groups bound by hydrogen bonds to the surface of diatom frustules [99]. All the samples presented a significant mass loss (from 3% for N_w to 22% for N_g) in the range from about 400 °C to about 700 °C, corresponding to strong exothermic peaks in the DSC curves. In fact, this loss may be due to the decomposition of organic matter of diatom biomass [74] containing molecules such as lipids and proteins [100,101]. In addition, for materials doped with titanium, this range coincides with the temperature range (400–480 °C), corresponding to the amorphous transformation of TiO_2_ into anatase form [102]. The fourth phase was distinguished in the temperature range from about 690 °C to 800 °C, with an endothermic effect and a mass loss corresponding to 16% for N_g, 20% Ti_15_g, 2% Ti_15_w and 2% for N_w. This phase was characterized by strong asymmetric DSC peaks and could be due to the decomposition of calcium carbonate and the accompanying carbon dioxide emission [103]. At the same time, for samples doped with titanium, this range also includes the transformation of the anatase form of TiO_2_ to rutile (690–750 °C) [102], which is also indicated by XRD results. A minor weight loss of the analyzed samples with an endothermic effect on the DSC curves was observed in the temperature range from about 800 °C to 1000 °C. This stage may be related to the removal of internally isolated hydroxyl groups [20]. It can be observed that the final residue was highest for purified biosilica and titanium doped biosilica samples (ca. 90% for N_w and ca. 68% for Ti_15_w), compared to biomass samples (ca. 44% for N_g and 50% Ti_15_g).

### 3.8. The FT-IR Spectra

**Figure 9:**
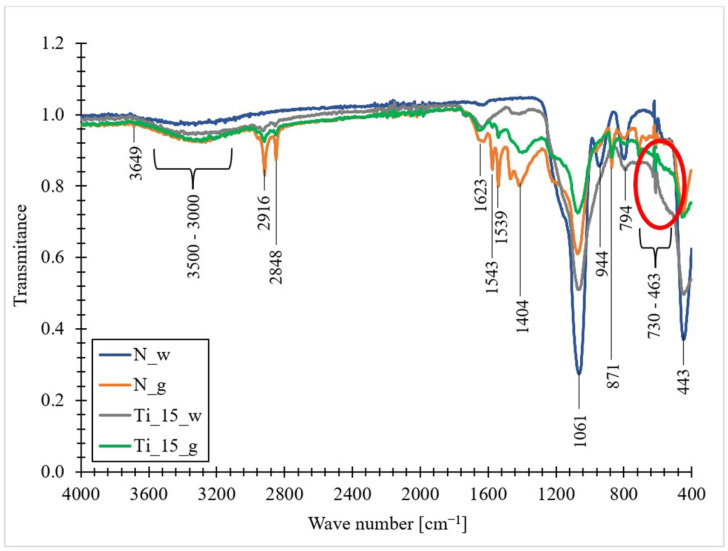
FTIR-ATR spectra of undoped biomass, titanium-doped biomass, pure diatomaceous biosilica and titanium-doped diatomaceous biosilica (N_g-undoped biomass; N_w-pure diatomaceous biosilica; Ti_15_g-the biomass doped with titanium ions with a concentration of 15 mg Ti/L; Ti_15_w-the biosilica doped with titanium ions with a concentration of 15 mg Ti/L).

Figure 9 shows the FTIR-ATR spectra of the obtained materials. In some regions the spectra of samples containing titanium differ from those that do not. The same situation occurs when comparing biomass samples with biosilica samples. These differences may result from the content of the doped element (when comparing doped and undoped materials), as well as the presence of an organic part (when comparing biomass and biosilica) in the material. By analyzing the FTIR spectra obtained, the presence of 12 characteristic signals in the form of peaks and two representative wavelength ranges can be observed. The band at 3649 cm^−1^ likely corresponds to the isolated hydroxyl groups [104]. A wide band from 3500–3000 cm^−1^ is related to the O-H stretching vibrations of molecular water with hydrogen bonds. It can also be associated with the presence of an N-H bond associated with the residues of biosilica precipitating proteins, which include long-chain polyamines and sylaphins, which were involved in the formation of diatom frustules [105,106]. The appearance of two sharp peaks at 2916 cm^−1^ and 2848 cm^−1^ likely corresponds to the C-stretching vibrations of the methyl and methylene groups (CH_3_- and CH_2_-), which are associated with lipids, and therefore are most visible in the biomass samples. The three bands at 1623 cm^−1^, 1543 cm^−1^ and 1404 cm^−1^ correspond with the aromatic stretching vibrations =C–C [107]. The sharp peak at 1539 cm^−1^ may be due to the presence of secondary amide groups [108]. The band at 1439 cm^−1^ corresponded with C–O stretching vibrations, and the band at 711 cm^−1^ was related to Ca–O bonds in the structure of CaCO_3_ [109].

Asymmetric stretching vibrations of Si-O-Si bonds reflect the most intense band located at 1061 cm^−1^. The band detected at 944 cm^−1^ is assigned to the stretching vibrations of the Si groups. Additionally, it is characteristic of marine diatoms [110] and is associated with their amorphous silica shell [111,112]. The sharp peak at 871 cm^−1^, detected in the samples containing the organic part, may be related to the occurrence of the C-H bending vibration [108]. The band that appears at 794 cm^−1^ is attributed to the symmetrical stretching vibrations of the Si bonds. According to Adamo [113], this band is characteristic of amorphous silica, such as opal-A. A wide band in the range of 730–463 cm^−1^, present in the spectra of titanium-doped samples, is attributed to the Ti-O stretching modes and the Ti-O-Ti bridge [114]. For pure TiO_2_, the peaks at 463 and 728 cm^−1^ are characteristic of TiO_2_ in the form of anatase. Additionally, the signal at 463 cm^−1^ is attributed to the Ti-O bond vibration in the TiO_2_ lattice, and the signal at 730 cm^−1^ is attributed to the Ti-O-Ti stretching vibration. [115]. The last band, occurring at 443 cm^−1^, is related to the Si-OH bending vibrations in the siloxane groups [105,111,113,116].

### 3.9. Photoluminescence Spectra

**Figure 10:**
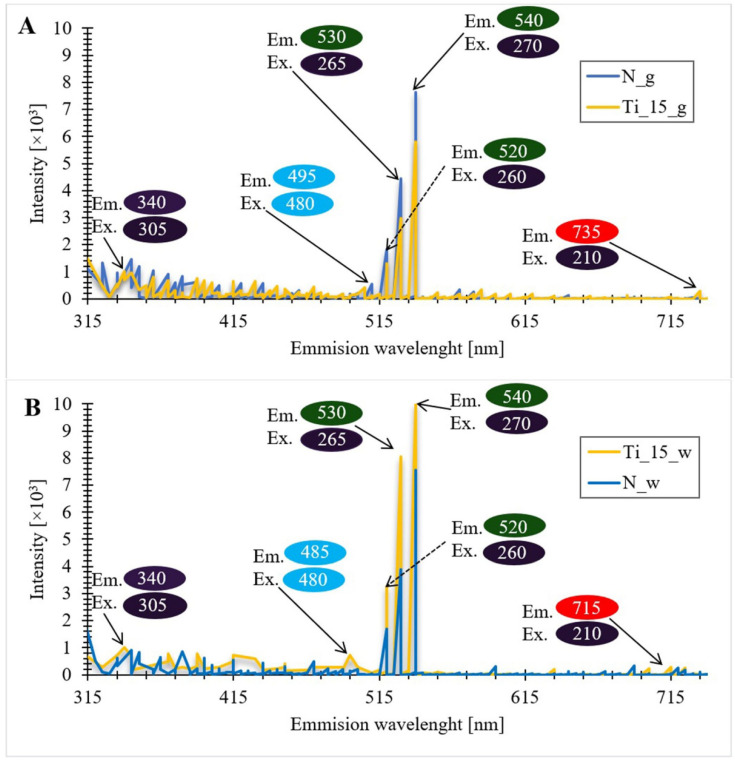
Photoluminescent spectra of undoped diatom biomass of the species *Pseudostaurosira trainorii* and biomass doped with titanium ions (**A**), as well as pure diatomaceous biosilica and biosilica doped with titanium ions (**B**) (N_g-undoped biomass; N_w-pure diatomaceous biosilica; Ti_15_g-the biomass doped with titanium ions with a concentration of 15 mg Ti/L; Ti_15_w-the biosilica doped with titanium ions with a concentration of 15 mg Ti/L).

When analyzing the obtained photoluminescence spectra (Figure 10), it should be noted that in all materials, three main types of photoluminescence activity (PL) can be distinguished.

The first type is associated with excitation and emission in the ultraviolet region (315–380 nm). In this region, the most intense photoluminescence appears in peaks with a wavelength of 340 nm under excitation of 305 nm. The second type is related to excitation-emission in a very narrow blue region of the visible spectrum (excitation at 480 nm and emission at 495 nm for Ti_15_g and 485 nm for Ti_15_w). The third type of photoluminescent activity is characterized by very intense emission in the green region (496–566 nm). In this region of the visible light spectrum there are three regions of high intensity: I-excitation at 260 nm and emission at 520 nm, II-excitation at 265 nm and emission at 530 nm, and III-excitation at 270 nm and emission at 540 nm. The fourth type of photoluminescence activity appears on PL spectra obtained for materials doped with titanium ions. This type is related to the emission in the red-light region (at 715 nm for doped biomass and at 735 nm for Ti-doped biosilica) and excitation at 210 nm.

The photoluminescent activity of diatomaceous biosilica in the ultraviolet region can be attributed to the fluorescence of organic residues embedded in the structure of the biosilica [117,118]. Additionally, in materials doped with titanium ions, photoluminescent activity in the ultraviolet region may be associated with self-entrapped excitations located on the TiO_6_ octahedron, resulting from the presence of the anatase form of TiO_2_ in the sample [119,120]. The intensity of the photoluminescence peaks assigned to titanium in the ultraviolet region is not high. This may be due to the SiO_2_ matrix effect and the dispersion and fragmentation of TiO_2_ particles in the SiO_2_ matrix [29].

The origin of the intense emission in the regions of blue and green visible light may be the result of Si-O surface defects, related to nanoporous silica [121], as well as non-bridging oxygen hole centers (^●^O-Si≡), self-entrapped excitons (-O-O-) and neutral oxygen vacancies (≡Si-Si≡) [122,123,124,125]. The photoluminescent activity of materials doped with titanium ions in the red region may be the result of surface defects related to oxygen vacancies or defects related to the presence of Ti^4+^ ions adjacent to oxygen vacancies [126]. Wu et al. [127] mentioned that the photoluminescence emissions observed in the visible range may be due to carrier concentration recombination between the valence band and the oxygen vacancies acting as donor levels. Interpreting the obtained photoluminescence spectra, it can be noticed that in the sample containing the biomass doped with titanium (A), there is a decrease in the intensity of signals in the regions of blue and green light, compared to the biomass without the doped element. This is probably the result of the suppression of the photoluminescent activity of titanium oxide with the organic layer of diatom cells. For biosilica doped with titanium ions (B), an increase in signal intensity was observed in blue and green light regions compared to pure biosilica. This is due to the fact that in the regions of the 460 nm and 530 nm band there are oxygen vacancies in the anatase form of TiO_2_, which strengthen the oxygen vacancies originating from silica [120,128]. TiO_2_ nanoparticles present a strong absorption in the UV which is reflected in the enhancement of the photoluminescence of the biosilica in the green region 520–540 nm. The nature of such an enhancement of the emission in the green region can be caused by the photoluminescence activity of the TiO_2_ nanoparticles in the narrow range of UV bands and activation of the oxygen vacancies of the biosilica, as well as by the fact that TiO_2_ in the anatase form also shows a broad emission in a green light diapason at UV excitation [129,130] and could enhance the green fluorescence from the biosilica.

## 4. Conclusions

The titanium doped diatomaceous biosilica, with a three-dimensional hierarchical structure and specific photoluminescence properties, was obtained as a result of the conducted experiments using the methods of metabolic insertion. The combination of the original three-dimensional structure and photoluminescent properties in the obtained material makes it an attractive source of solutions in the development of modern materials engineering. After a series of experiments related to the selection of the appropriate pH, initial titanium concentration, and optimal titanium/silicon concentration ratio, a material was obtained that proved to be a compromise between the amount of biomass grown and its titanium content. An initial titanium concentration 15 mg/L, pH value 8.9, and initial silicon concentration 1.2 mg/L (Ti:Si = 1:17.5) were defined as the optimal conditions for metabolic titanium insertion into diatom cells. The biosilica obtained by purification of the obtained biomass had a titanium content of 8.59% wt.

The study results of adsorption kinetics of nitrates, phosphates, silicon, and titanium by diatom cells from the culture medium are showing that the absorption of titanium is rated similar to the absorption of silicon (similar shapes of kinetic curves) and may suggest a similar mechanism of uptake.

Scanning electron microscopy images showed the morphology and three-dimensional structure of the titanium doped diatom frustules similar to that of undoped diatom frustules. SEM-EDX analysis also showed the even distribution of the doped element in the materials. The mass percentage of titanium in the purified biosilica was higher (8.59%) than in the biomass (6.57%). TEM analysis visualized the coverage of the surface and pores of the frustules by fragments of a thin coat in the state of irregular flakes containing ooid forms of the embedded nanoparticles, TiO_2,_ with dimensions of 10–20 nm. Additionally, titanium is also detected in the parts of the frustules not covered by flakes, that may be indicative of the possibility of the titanium partially incorporated into the structure of the diatom shell by isomorphic substitution of silicon atoms. However, the nature of the binding phenomenon between the resulting flake and the siliceous surface of the diatom frustules remains unexplained.

The XRD analysis of the diatomaceous biosilica doped with titanium subject to pyrolysis at 1000 °C indicated the transition of the silica and titanium oxides from the amorphous phase to its crystalline forms. Two crystalline forms of TiO_2_ (such as anatase and rutile) and SiO_2_ (such as quartz and tridymite) have been observed.

The thermogravimetric measurements of the obtained materials revealed the occurrence of five stages of mass loss, which were related to different forms of water bonding, i.e., to the physical bonding of the water molecule, to hydrogen bonds of vicinal hydroxyl groups, to free hydroxyl groups, as well as to the decomposition of organic matter of diatom biomass and carbon dioxide emission. In addition, the results of TG and DSC analysis suggest the occurrence of transformation of the amorphous form of TiO_2_ into anatase and, after exceeding a temperature of about 700 °C, into rutile form.

The obtained titanium doped biosilica is characterized by four types of photoluminescence activity: excitation and emission in the ultraviolet region; excitation and emission in the very narrow blue region of the visible spectrum; intense emission in the green region upon excitation in the ultraviolet region; and emission in the red-light region and excitation at the ultraviolet area.

Metabolic insertion of titanium into diatomaceous biosilica allowed the obtaining of new silica materials with interesting semiconductor and optoelectronic properties. Such materials can be used in modern technologies for the production of biosensors, optical devices, catalysts, semiconductors, efficient adsorbents, drug carriers or nanolithography matrices. The obtained material with a three-dimensional structure, perforated by rows of pores, can also be used as an excellent filter material for gel permeation chromatography for protein purification, or as an effective adsorbent material for filling chromatographic columns in liquid chromatography and SPE extraction.

## Figures and Tables

**Table 1:** Generalized set of cultivation conditions during the series.

	Series (I–IV)
Temperature [°C]	25
Light intensity [lux]	1500
Ilumination (fotoperiod) [h:h]	12:12
Photobioreactor volume [L]	25
Culture medium	Guilard’s f/2
Si precursor	Na_2_SiO_3_ × 5H_2_O
Ti precursor	C_16_H_36_O_4_Ti in 37% HCl_aq_
Culture period [days]	12

## Data Availability

The data presented in this study are available upon request.
